# A Smart Mirror for Emotion Monitoring in Home Environments

**DOI:** 10.3390/s21227453

**Published:** 2021-11-09

**Authors:** Simone Bianco, Luigi Celona, Gianluigi Ciocca, Davide Marelli, Paolo Napoletano, Stefano Yu, Raimondo Schettini

**Affiliations:** Department of Informatics, Systems and Communication, University of Milano-Bicocca, Viale Sarca 336, 20126 Milano, Italy; simone.bianco@unimib.it (S.B.); luigi.celona@unimib.it (L.C.); gianluigi.ciocca@unimib.it (G.C.); davide.marelli@unimib.it (D.M.); s.yu1@campus.unimib.it (S.Y.); raimondo.schettini@unimib.it (R.S.)

**Keywords:** smart mirror, affective computing, multimodal interaction, Raspberry Pi, deep learning, internet of things, Amazon Alexa virtual assistant, positive technology

## Abstract

Smart mirrors are devices that can display any kind of information and can interact with the user using touch and voice commands. Different kinds of smart mirrors exist: general purpose, medical, fashion, and other task specific ones. General purpose smart mirrors are suitable for home environments but the exiting ones offer similar, limited functionalities. In this paper, we present a general-purpose smart mirror that integrates several functionalities, standard and advanced, to support users in their everyday life. Among the advanced functionalities are the capabilities of detecting a person’s emotions, the short- and long-term monitoring and analysis of the emotions, a double authentication protocol to preserve the privacy, and the integration of Alexa Skills to extend the applications of the smart mirrors. We exploit a deep learning technique to develop most of the smart functionalities. The effectiveness of the device is demonstrated by the performances of the implemented functionalities, and the evaluation in terms of its usability with real users.

## 1. Introduction

People’s emotions can change a lot in a short span of time. Sometimes it’s possible to see those changes in a matter of days or even hours. The problem is when those changes end up negatively, resulting in lower productivity and possibly impacting relationships such as a lover, work colleague, or friend. Monitoring those emotions can be really challenging since it is uncommon to own a specific device for that kind of purpose. A good solution is to transform an object that everyone owns such as a mirror and use it for monitoring the user’s emotions and, at the same time, offer features for emotional support in a *smart* way. Nowadays, a mirror is a common object that everyone owns. Whether it’s stationed in the living room, bedroom, or bathroom, a mirror is something that most people use everyday. Thus, it seems obvious to transform a normal mirror into a smart mirror with the capabilities of detecting and helping the user emotionally, both in the short- and long-term. The idea of a smart mirror is not something new, and the conception of this idea dates back to the late 1990s and early 2000s with science-fiction movies. Even though it started as a “Do It Yourself” (DIY) project around 2013–2014, the first known smart mirror was built by Michael Teeuw back in 2014 using a Raspberry Pi 2 (MagicMirror—http://michaelteeuw.nl/tagged/magicmirror (accessed on 15 October 2021)). The original project moved forward with a second version of the Magic Mirror (MagicMirror^2^—https://magicmirror.builders/ (accessed on 15 October 2021)). In the same year that Magic Mirror was released, Toshiba presented their own concept of a “smart mirror” at CES [1]. Those mirrors, using a reflective screen, were able to display not only general information such as weather, news, and email, but also personal data like calorie consumption, heart rate, steps, etc., obtained from connected devices. With time, the concept of smart mirror evolved, and nowadays, it is easy to create a smart mirror with those basic features as more specialized types of smart mirrors started to emerge. Based on the field of use, smart mirrors can be classified into: general purpose, medical, fashion, and hotel. **General-purpose smart mirrors** are those built exclusively for everyday use. These mirrors are usually able to display general information such as news, weather, time, calendar, reminder, and alarms. The latest mirrors introduced more complex features (e.g., email reader, media player, browser, etc.) and security features (e.g., facial and voice recognition). **Medical smart mirrors** are an advanced version of the general purpose mirrors. They are in fact capable of displaying general information, but also medical features such as facial expression detection, emotion detection, skin problem detection, body pose detection, posture detection, etc. Furthermore, they can include related tips and tricks to help the user to solve the discovered problem. **Fashion smart mirrors** commonly include the virtual try on technology. The goal of these mirrors is to enable customers to try on products such as clothes, shoes, cosmetics, or jewelry using their camera and Augmented Reality (AR). **Hotel smart mirrors** comprise specific features for the hotel in which they are currently installed. These features usually allow the user to access room amenities, such as room temperature or humidity, room devices (e.g., TV or lighting), pay for additional services, and book and receive notifications from the hotel staff. These mirrors are not sold to the general public, and not many hotels are currently adopting this technology.

Table 1 and Table 2 summarize the main functionalities and technologies of the smart mirror in the literature, and those commercially available. With respect to the general purpose smart mirrors, we can see that most of them share the same basic functionalities. They are mostly designed to support the user in managing and collecting information, and to ease the control home devices. The interaction is mostly based on voice commands and no authentication protocols are included. Fashion mirror are very specialized and often include some form of augmented reality to engage the user and recommend or try virtual clothes and accessories. The mirrors targeted to the medical or health fields include some form of direct or indirect health monitoring often using physical sensors. Finally, for the commercial mirrors, we can see that they provide many functionalities for services in the cloud. Little information can be found about the underlying technologies.

From this analysis, we see that smart mirrors have either basic functionalities or are too specialized. Here, we propose a general purpose smart mirror that incorporates a heterogeneous set of functionalities that can help users in their everyday lives. The mirror is designed to include both common functionalities as well as advanced functionalities specifically targeted for monitoring the well-being of the user. These functionalities are often found in medically targeted smart mirrors. Moreover, a suitable authentication protocol should be considered to protect the privacy of the user and provide access to the sensible information. As far as we are aware, no prior work on smart mirrors tackle all these issues in a satisfactory way. Thus, due to the limited functionalities of existing general-purpose smart mirrors, in this paper we illustrate the design of a novel prototype of smart mirror device that is capable of detecting and identifying the user, assessing their current emotional state through analysis of the user’s face and voice features, and interacting with the user in different modalities. Our smart mirror offers a dynamic interface that displays not only the current data, but also information inferred from the analysis of stored long-term data. This is especially important in the case of the user’s emotions. From the comparison of the current and past emotional states of the user, the mirror can suggest ways to mitigate negative emotions. From this perspective, the use of our smart mirror to detect emotions and assist the users can be seen as a form of “Positive Technology”; that is, the use of technology for improving the quality of one’s personal life [2,3].

In summary, the main contributions of this paper are:

- The design of a general purpose smart mirror that is easily extendable using ad-hoc modules and the integration of Alexa’s Skills;
- The integration of our deep learning-based modules for the user’s identification, facial attributes estimation, and emotional recognition from visual data;
- The design and implementation of the deep learning-based modules for both speaker identification and emotional recognition from audio signal;
- The evaluation of the audio-based recognition modules under the speaker-dependent and speaker-independent scenarios;
- The definition of a double authentication protocol for a more secure user identification;
- An extended system usability survey to evaluate the final system and gather user feedback for future improvements.

**Table 1:** A summary table of main features and technologies of smart mirror prototypes in literature. Field indicates intended use of mirror: (G)eneral, (M)edical, and (F)ashion.

Ref.	Field	Features	Technologies
Our	**G**	General information, email notifications, face identification, speaker identification, emotional states monitoring, Joybar, virtual avatars, multimedia, Alexa’s Skill integration	face recognition, voice recognition, speech recognition, emotion recognition
[4]	**G**	General information, email notifications, daily schedules, graphical keyboard	speech recognition
[5]	**G**	General information, multimedia, two languages, mobile app.	-
[6]	**G**	General information, road traffic, voice commands	face detection, speech recognition
[7]	**G**	General information, voice commands	speech recognition
[8]	**G**	General information, web browsing, face identification, mobile app.	face recognition, speech recognition
[9]	**G**	General information, face identification	face recognition
[10]	**G**	General information, 3D graphics, voice commands	speech recognition, face tracking
[11]	**G**	General information	eye tracking
[12]	**G**	General information, Alexa’s Skill integration	speech recognition
[13]	**G**	Track faces, video playback	face detection
[14]	**G**	3D visualization	gestures recognition, augmented reality
[15]	**G**	General information, voice commands, face identification	face recognition, speech recognition
[16]	**G**	General information, voice commands, home device control	speech recognition
[17]	**G**	General information, mood detection	emotion recognition
[18]	**G**	General information, voice commands	speech recognition
[19]	**G**	General information, voice commands, face identification	face recognition, speech recognition
[20]	**G**	General information, home devices control, chat assistant, face identification	Speech recognition, Face recognition
[21]	**G**	General information, voice commands	speech recognition
[22]	**G**	General information, voice commands	speech recognition
[23]	**G**	General information, voice commands	speech recognition
[24]	**F**	Fashion recommendation	gesture recognition, augmented reality
[25]	**F**	Virtual try-on, 3D visualization	virtual reality, body tracking
[26]	**F**	Virtual try-on, 3D visualization	body tracking, augmented reality
[27]	**F**	Makeup recommendation	face detection
[28]	**F**	General information, face identification, emotion detection, clothes recommendation	face recognition, emotions recognition
[29]	**M**	3D scanning, face identification	facial expressions detection, face recognition, 3D face reconstruction
[30]	**M**	3D graphics, fatigue detection, user fitness, digital measurements	face detection, 3D face reconstruction, face tracking
[31]	**M**	Therapy exercises	body pose tracking
[32]	**M**	Mood detection	emotion recognition
[33]	**M**	General information, health monitoring, BMI calculator (w/ sensors), face identification	face recognition
[34]	**M**	General information, smart posture assistant, face identification	face recognition, Posture recognition
[35]	**M**	General information, face identification, health monitor (w/ sensors)	face recognition
[36]	**M**	General information, music therapy	emotion recognition, face detection
[37]	**M**	General information, color therapy	emotion recognition, face detection
[38]	**M**	General information, face identification, music therapy	face recognition, emotion recognition
[39]	**M**	General information, music therapy	emotion recognition, face detection

**Table 2:** A summary table for commercial smart mirrors. Field indicates intended use of mirror: (G)eneral, (F)ashion, and (H)otel.

Ref.	Field	Features	Technologies
[1]	**G**	General information, multimedia, cooking helper	hand gesture recognition
[40]	**G**	Bill payment, TV, pay per View, screen for PC applications	–
[41]	**G**	General information, health tracker, TV, weather, photos viewer, social networks feeds, mobile app	–
[42]	**G**	General information, weather, multimedia, mobile app	face recognition, hand gesture recognition
[43]	**G**	General information, weather, mobile App	–
[44]	**F**	Clothes management and recommendation, mobile App	augmented reality
[45]	**F**	Makeup recommendation, personal improvement plan	skin imperfection detection
[46]	**H**	General information, weather, transport information, events, shop, social network integration, mobile app	–
[47]	**H**	General information, hotel services, local transportation	based on IBM Watson AI

The paper is structured as follows. In Section 2, we comment on recent works related to smart mirrors, both academic and commercial. In Section 3, we describe the design of our smart mirror, from the functionalities to the hardware and software implementation. In Section 4, we report some quantitative results on some developed modules incorporated into the mirror. We also report on the results from a subjective study on the usability of the whole system. Finally, Section 5 concludes the paper.

## 2. Related Work

The popularity of smart mirrors rapidly increased, and many articles on the subject are featured in the literature. Most of the existing work describes the design of a general type of smart mirror that includes only simple basic functions such as news, weather, alarm, time, etc. However, there are works involving smart mirrors developed for medical or fashion purposes. In this section, we briefly review the state-of-the-art in smart mirrors both in the research/prototype stage and commercial ones. For a summary of the functionalities and technologies of these smart mirrors, see Table 1 and Table 2.

### 2.1. Smart Mirror Prototypes

General purpose smart mirrors can be grouped primarily by the input sensors with which they are equipped. Vision-based smart mirrors are equipped with an RGB camera. They commonly activate when they recognize the framed user and show contents dedicated to the variation of the user’s expressions [9,11,13,17,48,49].

Several smart mirrors are equipped not only with visual input but also with audio input. Most of these mirrors use voice control algorithms to access the mirror functions [7,18,23], others exploit the combination of audio-video recognition to obtain more robust security protocols [8,15]. More complex mirrors take advantage of technologies such as: Global System Module (GSM) to send emergency calls [4]; Ambient Intelligence (AMI) to control a set of custom services [5]; and Augmented Reality (AR) to provide education and entertainment services [14].

In the medical field, smart mirrors can improve both clinical and at-home healthcare. Several proposals rely on the analysis of the face and facial expressions for daily personal checkups [29,30,32,50]. Some mirrors complement the diagnosis with alternative medicine treatments such as music therapy [36,38,39] and color therapy [37].

More sophisticated mirrors can link with sensors, wearable devices, or other smart products to collect physiological signals. In [33], the authors proposed a smart fitness mirror to monitor the user’s health. Load and ultrasonic sensors were used to detect the user’s weight and height, electrode plated for bio-electrical impedance analysis, finally, an IR temperature sensor to measure the user temperature. Bluetooth and LoRa (short for Low Range) modules were used in [51] to measure basic information such as a temperature, and humidity of the room.

Some smart fashion mirrors include recommendation systems. For example, in [28] to suggest the ideal outfit basing on the user’s mood, or in [27], the makeup that best suits the user’s face. The use of Augmented Reality (AR) and Virtual Reality (VR) in smart mirrors to improve the customer experience is increasingly widespread. In fact, both AR and VR can help make retail stores more interactive and the online experience more real [24,25,26].

Smart mirrors are also exploited for monitoring the users as smart healthcare systems. The device is used to record the user’s health conditions, remind about medications, suggest exercises, and, generally, monitor the well-being of the user. The paper by Silapasuphakornwong et al. [52] is a recent example of this kind of smart IoT device. The authors designed a smart mirror that can talk and have a conversation with elderly users so that they do not feel lonely. It monitors their mental well-being by analyzing their emotions and behavior. Another example is the LUX system [53], which analyzes user sentiment and manages sentiment feedback by exploiting speech-to-text, NLP, and deep learning techniques. Currently, LUX is able to manage Korean text and provides feedback in the form of reciting wise sayings, empathizing, and playing music.

### 2.2. Commercial Smart Mirrors

This section is dedicated to commercial smart mirrors that are developed outside the research laboratory and the DIY community. The smart mirror is still a growing market, and companies are still developing their own product. However, we can find some smart mirror products on the market. There are few general-purpose commercial smart mirrors. Ekko [42] offers basic features such as general information and personalized profile and can be controlled with hand gestures. Griffin Technology [43] developed the Connected Mirror, a smart mirror showing general information (time and weather, phone notifications), and updates from other Griffin devices. Toshiba [1] created a mirror that can help the user prepare recipes and work as a personal fitness monitor through a connection with a smartphone. Chakra Groups [41] released a smart mirror offering health-related features (e.g., tracking for weight, calories, sleep, exercise) by connecting to Apple Health or Fitbit.

As part of the smart mirror for fashion category, Memories [44] uses a multilayer AR and AI engine. It allows a realistic and personalized augmented reality experience to try on clothes with colors and patterns of the user’s choice. Instead, Hi-mirror Plus [45] was an intelligent makeup mirror that could detect and analyze the condition of the user’s skin and offer advice to hide imperfections.

Anna smart mirror [46] was developed for hotels and, through the recognition of hand gestures, allows booking transport, viewing general information, integration, and management of social web via a web app. Philips [40] produced a smart mirror/TV that can be installed in hotels and helps the customer pay their bills or pay per view movies. In 2017, Panasonic [47] unveiled Digital Concierge, a smart mirror powered by IBM Watson advanced functionalities.

More recently, CareOS [54] presented Themis, a small smart mirror that can track the user condition by collecting data from different sensors, such as a high-quality RGB camera, an IR temperature sensor, and a UV light for skin analysis.

## 3. The Proposed Smart Mirror

The proposed smart mirror provides the user with an interactive interface that can be used comfortably in the home environment. The mirror can acquire information relating to the user’s emotional state and react accordingly. Users can access personal notes, reminders, calendars, news, weather information, and more. The following sections describe how the mirror interacts with external stimuli and what technologies (hardware and software) are used to develop the prototype.

### 3.1. Functional Requirements

The smart mirror is normally in standby mode until a subject standing in front of it acquire the *active user* role and trigger the mirror. If there are multiple subjects in front of the mirror, the selection of the subject to consider as the active user is based on two criteria:

- Face measurement. The subject whose detected face has the highest resolution is selected (usually the user closest to the mirror).
- User identification. The subject whose identity is known since it was previously registered is chosen. In the case of multiple registered users, the face measurement policy is also applied to them.

The presence of an active user will activate the display modules. Furthermore, the user will be able to access and interact with all the built-in functionalities.

**Figure 1:**
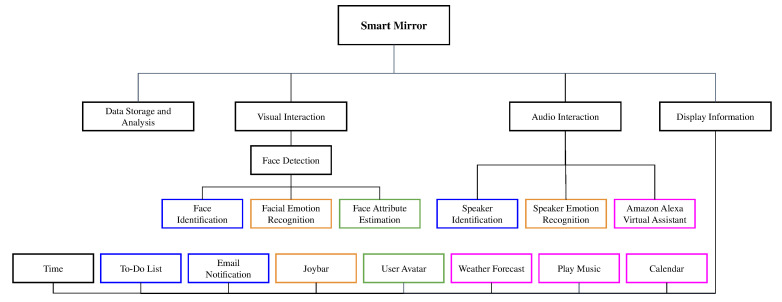
Functional requirements of the proposed smart mirror. Color of boxes indicates relationship between processing modules and information displayed by different applications. For example, to implement user-specific applications, user authentication through face and speaker recognition are required. Applications such as weather forecast, calendar, and music player depend on integration of mirror with Alexa Skills.

Figure 1 shows the set of functionalities that are implemented in our smart mirror. The color of the boxes indicates the relationship between the processing modules and the applications. Through visual and audio stimuli, the user can interact with the mirror and have access a variety of information. The two modalities support and complement each other in different tasks. For example, they are used for user identification: the visual stimuli is the main mode for user recognition, while the audio stimuli can be used as secondary identification module to make the recognition more accurate. Moreover, the visual stimulus is used as part of the emotion recognition process and is the main source for the face attribute detection exploited in the creation of the user’s avatar. The audio stimuli is also used for emotion recognition. As in the case of the identification, the exploitation of the two modalities allow us to capture more cues on the user’s emotion that cannot be achieved by leveraging only one modality. Finally, the audio is the main stimuli for the interaction with all the Alexa-based services.

#### 3.1.1. Visual Interaction

When the mirror is in standby mode, the face detection module is executed in the background. It is a fundamental function of the mirror, which also triggers the subsequent modules, namely, the face identification, the facial emotion recognition, and the face attribute estimation modules.

##### Face Detection

The face detection module consists of two steps: localization of the face in the current frame, and alignment of the cropped face region. For both steps, we based our implementations on the algorithms provided by the Dlib library [55]. The face detector is built using a sliding window detection scheme based on the Histogram of Oriented Gradients (HOG) as a descriptor combined with a linear SVM as a classifier. An image pyramid is used to detect faces at different resolutions.

For the success of the subsequent modules, we need to detect faces in a frontal or near-frontal pose. For this reason, we preferred a HOG-based to a deep learning-based face detector. The first, in fact, although less robust, is sufficiently accurate and very efficient.

Regions corresponding to detected faces are cropped to obtain facial images. Hence, each of these facial images is processed to align facial features to a canonical position. This step is necessary to reduce the variability between different facial images due to pose differences. We exploit a facial landmarks based alignment method to fix the position of both eyes and chin within each facial image [56]. The aligned facial image is then scaled to a fixed shape of 224×224 pixels.

##### Face Identification

The face identification module is activated after faces are detected and aligned. The module consists of a ResNet-50 pre-trained on frontal faces and specifically trained for face identification. The network is used to extract features as a representation of the user’s face and save them as numeric vectors, including a unique ID that is assigned to them. These features and associated labels are fed to a *k*-Nearest Neighbors (*k*-NN) algorithm to classify new faces based on the nearest *k* representation from the given face representations. This procedure allows us to easily extend the set of the identities recognized by the mirror without requiring a retraining of the network in an end-to-end fashion. The features used for classification of the identities are collected during the registration phase. Since different acquisition conditions (e.g., room lightning, accessories worn, and facial expressions, etc.) may influence the accuracy of the recognition, during the registration phase, multiple representations of the user are captured by asking the user to rotate their face.

##### Facial Emotion Recognition

**Figure 2:**
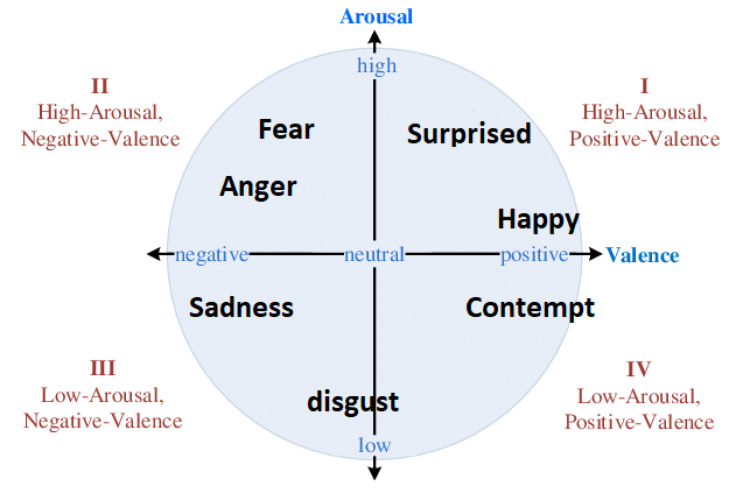
Two-dimensional valence-arousal space for emotions trained in model.

Facial emotion recognition is used to monitor common facial reactions to recognize if a person is experiencing a positive emotion if they are for example, smiling, or a negative one if they are frowning instead. We design this module to capture emotional state in terms of emotion categories as well as continuous valence/arousal space (see Figure 2). Following [57], we train a ResNet-50 using multitask learning on the 400,000 facial images of the AffectNet dataset [58]. The resulting model is capable, given a facial image, to predict one of the following 10 emotion categories: anger, contempt, disgust, fear, happiness, neutral, none, sadness, surprise, uncertain. Moreover, two scalar values are produced that correspond to the valence and arousal scores. These values are used to display the user’s emotional level in the joybar (see Section 3.1.3).

##### Face Attribute Estimation

Apart from recognizing user emotions, the system can also estimate facial traits such as hairstyle and color, skin color, and worn accessories (e.g., glasses and hats). The model used for face attribute estimation is the one proposed in [59]. It is based on a ResNet-50, whose predictions are conditioned by a layer that takes into account the correlation between the attributes. The model is trained using a binary cross-entropy loss on the CelebA dataset [60]. This dataset contains over 200,000 images of 10,000 different identities and a list of 40 attributes that describes general traits for each facial image (see Table 3 for details). These attributes are used to create an avatar with the user’s detected facial traits.

#### 3.1.2. Audio Interaction

The audio interaction is mainly intended to complement and support the visual one. Audio interaction is only required during the facial identity registration phase but optional later. Currently, the mirror offers the possibility of complementing visual identification (face identification) with vocal identification to increase the level of security. This allows us to perform the user authentication in a more robust way and protect personal information. Both the visual identification and audio identification must agree to grant the user access to the mirror. If the authentication process fails, the user is asked for another authentication try. The audio interface also includes an emotion recognition module. This, together with facial emotion recognition data, could lead to an increase in emotion recognition accuracy. Furthermore, the Amazon Alexa virtual assistant is implemented to guarantee the user access to smart services.

##### Speaker Identification

Given that the proposed mirror gives access to highly sensitive information (such as personal notes, emails, online banking), we decide to combine speaker identification with face identification. This extra layer of security adds and integrates with the previously illustrated face identification module. Speaker identification takes place in two phases: registration and authentication. During the registration phase, the user repeats three times the same passphrase, and the three audio signals are then fed to an embedder that computes the three feature vectors. The average feature vector of the three feature vectors is saved to represent the registered user. The method is based on Mel-Frequency Cepstral Coefficient (MFCC) features that are first extracted from the raw audio signal. MFCCs are computed in a sliding window fashion by using a Hann window of width 25 ms and step 10 ms, taking 512 FFT of the signal, and considering 40 Mel filter-banks. The ResNet-34 proposed in [61] is then used as embedder to encode the audio features into a feature vector. The CNN model is trained on the VoxCeleb1 dataset [62] by processing 3 s utterances for discriminating among 1251 speakers. At inference time, the classification layer of the embedder is removed, and the activations of the penultimate fully connected layer, obtained by processing the entire utterance, are used as feature vector. The identification is done by comparing the feature vector of the uttered passphrase with those stored in the system using a *k*-NN classifier. The authentication phase is implemented as soft identification: it verifies whether the speaker has the same identity as the user recognized by the face identification module.

##### Speaker Emotion Recognition

Facial expressions and gestures tend to be the most understandable form of emotional communication, but they are also most easily controlled in response to different social situations when compared to the voice. Thus, in the proposed mirror, we also include a Speaker Emotion Recognition (SER) module. The SER involves the analysis of the speech signal to identify the emotion based on characteristic features such as pitch, formant, and phoneme. The method used for speaker emotion recognition is the same as the one previously presented for speaker identification. Given an input audio signal, the MFCC features are extracted and input to the ResNet-34 architecture trained for emotion categorization. Given the reduced cardinality of the currently available datasets and to allow the model to be able to generalize better, the model is trained on the combination of five different datasets which are CREMA-D [63], EMO-DB [64], SAVEE [65], and TESS [66]. The characteristics of the datasets used are summarized in Table 4. The seven emotions that are shared by the considered datasets and that are currently recognized by our mirror are: anger, disgust, fear, happiness, neutral, sadness, and surprise.

##### Amazon Alexa Virtual Assistant

We incorporated into the mirror several functionalities based on Amazon’s Alexa. We design the Alexa virtual assistant module to extend the interactive capabilities of the mirror allowing us to make the mirror smarter. Thanks to Amazon’s Alexa it’s possible to achieve a grade of artificial intelligence that can boost and facilitate the user’s interaction with the mirror. In particular, the system is not only able to start conversation if certain conditions are met, answer generic questions, or follow up simple conversations, but it is also possible to create customized skills that covers more specific questions or requests. In fact, the Amazon Alexa virtual assistant can play audio, control the smart home, answer generic questions, and engage services to keep the user organized, informed, safe, connected, and entertained. Apart from general Alexa skills, we develop three custom skills: coordinate the registration process of a new user; ask if the user has completed specific daily activities; ask and play relaxing music if the user is currently not feeling well. The integration of Alexa and the modularity of the graphical user interface (based on widgets) allow the mirror to provide a satisfying user experience. Above all, it is possible to use and customize the services available to the mirror.

#### 3.1.3. Display Information

**Figure 3:**
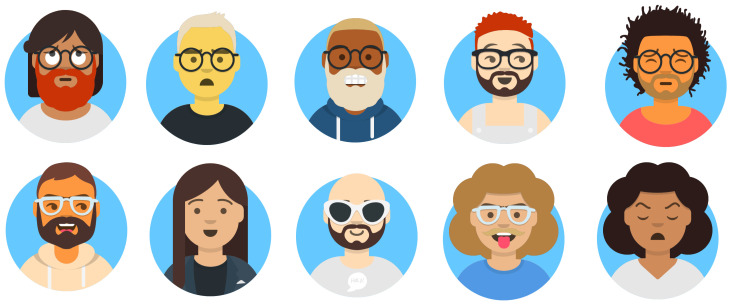
List of possible avatars that can be created with module Avatars.

The display monitor is the principal tool for showing information and mirror status. During the standby mode, only the time is displayed. After user authentication, several personal information are shown, such as the local weather forecast, emails, to-do list, calendar, and further widgets. The list of face attributes returned by the face attribute estimation module together with the emotion recognized by the face emotion recognition module is used by the Avatar module based on the Avatars library [67] to create an avatar that represents the current user. The choice of an avatar to represent the user’s face makes the system more attractive [68]. Furthermore, avatars were demonstrated to be very effective for therapeutic purposes [69,70]. In the proposed mirror, we could make the avatar smile or make funny to positively stimulate the user. Figure 3 reports a list of sample avatars with different peculiarities.

**Figure 4:**
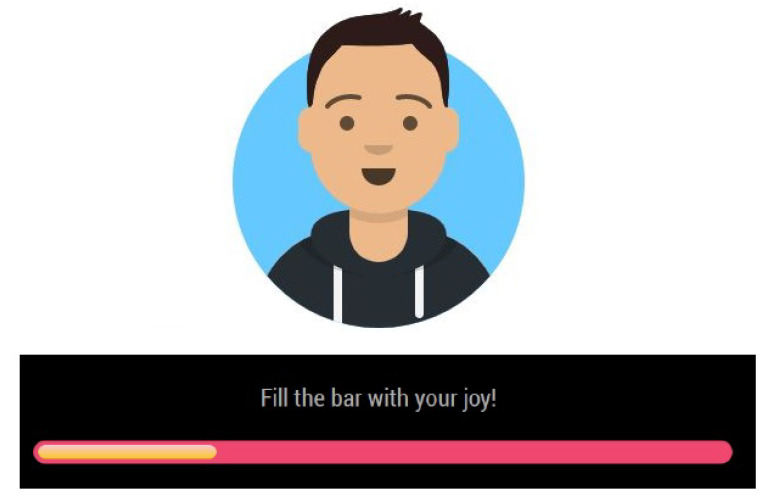
Example of user’s avatar with his joybar.

The user’s avatar is showed together with a joybar that measures the current user’s valence gathered from the face emotion recognition module. Figure 4 shows the combination of avatar and joybar on the user’s page. The display monitor is also responsible for showing information and feedback from the various active modules.

#### 3.1.4. Data Storage and Analysis

Most of the mirror features are based on on-the-fly data generated during user interaction. However, there is some information that need to be stored in the system, they can be recovered and analyzed at the appropriate time. The features and identities of the enrolled users are stored by the system and used for identification. Also, user’s preferences are stored in the system. Monitoring the user’s long-term emotional state is helpful in understanding his emotional progress and possibly diagnose disorders. For example, the emotional symptoms of distress can be traced back to long periods of depression/sadness, anxiety, and anger. To implement the previous feature, we store the emotional information that is automatically gathered each time the user interact with the mirror. Stored data can be exploited to create plots that represent the overall emotional progress of the user for a specific time frame, and those data can be analyzed to discover issues and improve the user’s emotional stability.

### 3.2. Technology Deployment

This subsection discusses the hardware and software technologies used in the building of the smart mirror system.

#### 3.2.1. Hardware

**Figure 5:**
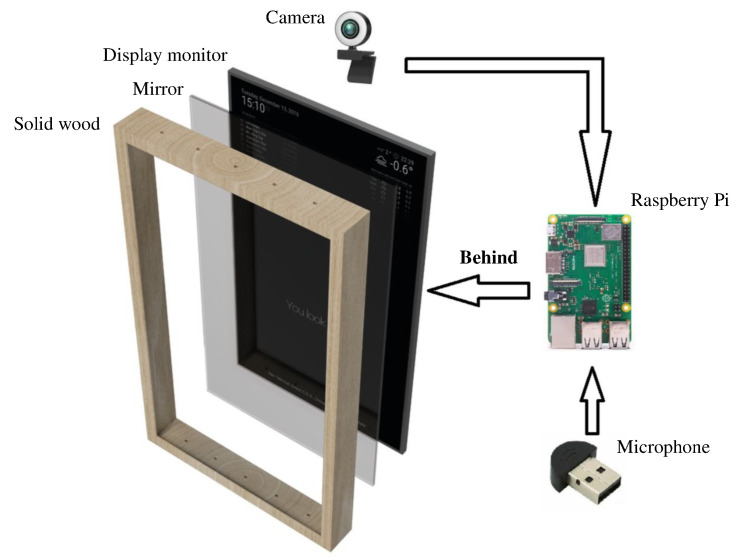
Hardware components of proposed smart mirror.

As illustrated in Figure 5, the smart mirror consists of five main hardware components:

- Display monitor: 27 Inch LCD monitor is used as display set.
- Micro-controller: Raspberry Pi 3B, one of the most popular single-board computer, for the role of the client.
- Camera and Microphone: 720P WebCam and Micro Microphone are employed to fulfill the visual- and audio-based functionalities.
- Mirror: A two-way surface with reflector surface properties is chosen.
- Frame: A solid wood frame box is built to cover internal components and place the display monitor.

The micro-controller is connected to the internet for data fetching and browsing. It is also linked to a serverless scalable service, Amazon Web Service (AWS) Lambda, to host and run the code for the Alexa Skill Service and an external server to offload the heavy computing and data storage. Micro-controller is Raspberry Pi 3 model B, a single-board based on Linux Operating System. The board has 64 bit CPU at 1.2 GHz, 1 GB of RAM at 900 MHz, WIFI, and Bluetooth. The Raspbian Stretch is the installed operating system. The board offers many I/O ports including Audio Jack 3.5 mm, 4 USB ports, GPIO, LAN, and HDMI. The LCD monitor is connected to the board through the HDMI port. USB ports are instead used to link both camera and microphone.

#### 3.2.2. Software

The smart mirror software is written in Python 3.8, and it is deployed in a Docker container. OpenCV and Pyaudio are exploited to capture the video frames and the audio signal, respectively. The client-server architectural model is implemented in Flask and then deployed using Waitress as Web Server Gateway Interface. Amazon Voice Service (AVS) is the cloud based service that allows to integrate Alexa features into the smart mirror. The interaction between Alexa and the Raspberry is handled by the avs library [71]. The library was modified to send the audio signal to the internal audio interaction module and not just to Alexa. MagicMirror2, one of the most popular open-source DIY smart mirror projects, is used as a starting point for providing the smart mirror with basic utilities. Both the avatar and the joybar are two additional modules that we designed for our mirror. The main software components in the proposed system are the following:

- The **Visual-Audio Manager** is responsible for the visual/audio inputs and outputs, including the initial interaction with Alexa, recording of audio, and capturing of visual frames.
- The **Graphical User Interface Manager** controls the information displayed in the mirror, including the user’s avatar, messages, joybar, and additional widgets.
- The **Data Processing Manager** handles and computes results from the given input for both visual and audio modules, including storing the data in the appropriate database.

**Figure 6:**
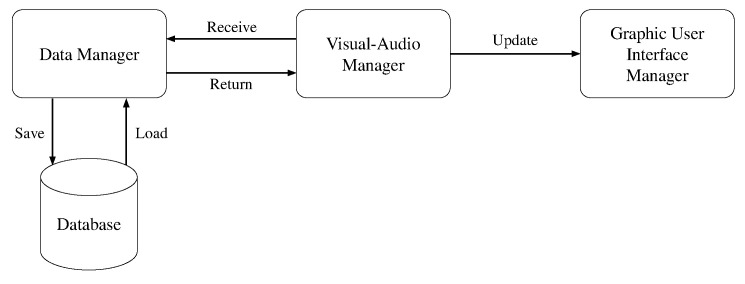
A simple diagram showing relationship between each software component.

The relationships between the three software components are shown in Figure 6. The Data Processing Manager receives the *raw data* (i.e., the audio signal and the video frames) from the Visual-Audio Manager and returns it the *computed data* (e.g., the predicted user’s emotion). The Data Processing Manager also interacts with the Database for storing and retrieving data. Finally, the Visual-Audio Manager sends the updates to the Graphic User Interface Manager for displaying information.

##### Visual-Audio Manager

**Figure 7:**
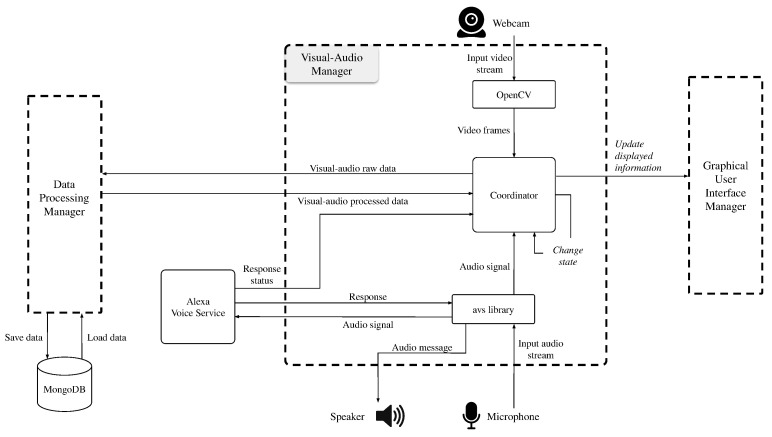
Components and interactions of Visual-Audio Manager in detail.

The Visual-Audio Manager coordinates the interaction with the user and the other software components. It can be considered the core module of the whole system. To better understand it, we graphically show its parts and interactions in Figure 7.

The main component of the Visual-Audio Manager is the Coordinator. It is in charge of handling the input-output streams for both the visual and audio signals. In the input data flow, the Coordinator acts as an intermediary for the Data Processing Manager. It receives the video frames acquired with the Webcam, and the audio signal recorded through the Microphone. It then forwards the Visual-Audio raw data to the Data Processing Manager, which returns the processed data. The Coordinator is also in charge of avoiding overloads and conflicts. This problem can arise due to (i) the availability of only one camera and one microphone, (ii) the presence of asynchronous services (i.e., Alexa Voice Service). Whenever a feature requires one of these devices, the Coordinator changes status to “locked” and blocks access to the resources until the devices’ proprietary function unlocks it. Finally, the Coordinator manages determines which screen (or page) has to be displayed from the Graphical User Interface Manager.

The input audio stream is also intercepted by the avs library. The avs library involves several components, namely the Voice-engine, the Alexa Voice Service (AVS), and the Alexa skill. The Voice-engine is used to capture the audio input. Since each Alexa skill is related to the pronunciation of a specific keyphrase, the Voice-engine can exploit the voice acquired with the microphone for user commands or a prerecorded audio file for automatic commands. The user’s voice is used to initialize a conversation with Alexa through keyword detection. This method involves external libraries: Snowboy and Hotword Detection. Thanks to these two libraries it is possible to train a model with a specific keyword for activating Alexa every time the user says that keyword. The audio file, on the other hand, is used to automatically initialize the conversation when a certain system condition occurs. An example of a condition is the authentication of the user by the system, another example of a condition is the lowering of the emotional valence below a certain threshold.

Alexa Voice Service acts as an intermediary between the Voice-engine and Alexa Skill. In fact, Alexa Voice Service processes the input audio stream eliminating the silence portions at the beginning and end of the recorded speech, and subsequently receives and plays the response from Alexa Skill. The received audio message is saved as a temporary file, and then eliminated by the operating system. Alexa Voice Service also sends a Response status to the Coordinator to manage the change in status of resources. Finally, Alexa Skill receives the processed audio signal and returns the response. If the key phrase is included in the Alexa Skill list, it will be processed; an exception is thrown away otherwise. In the proposed system, Alexa is used to determining the user name during the registration phase, reminding the activities to be carried out, and playing music audio in streaming.

##### Data Processing Manager

The Data Processing Manager contains most of the smart functions of the mirror and specifically all those relating to recognition from both video and audio streams. These functions are the most computationally expensive. Two components, one for the recognition from the video signal and the other for the recognition from the audio signal, are implemented. Each component executes one or more software modules that process a data stream.

For the visual data, given the video frames, the face detection pipeline is first executed, and then the identification, expression recognition, and attribute estimation modules are called on the facial images. For the audio part, the MFCC representation are first extracted using the Librosa library, and then this representation is sent to the speaker and emotion recognition modules. The results of the previous modules (the processed data) are returned to the Visual-Audio Manager.

For the storage of persistent or long-term data, we use MongoDB given its popularity, simplicity, and easy integration with the Python environment.

##### Graphical User Interface Manager

**Figure 8:**
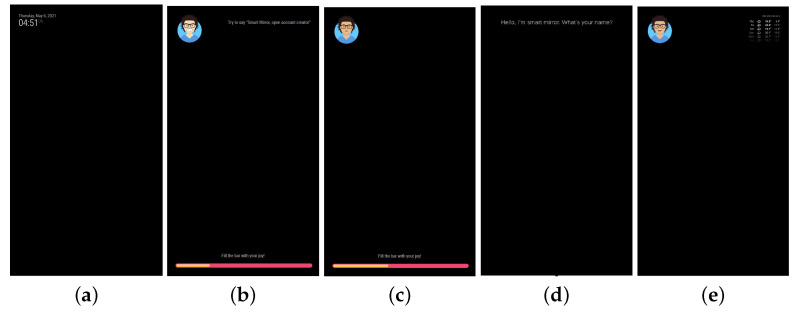
Pages that user can view. (**a**) Standby. (**b**) User not identified. (**c**) User identified. (**d**) Registration. (**e**) User’s personal page.

This component manages the Graphical User Interface or GUI. Figure 8 shows the relevant screens (or pages) displayed to the user during interaction:

- **Standby.** It is displayed when no user is detected in front of the mirror. Whenever this page is active, a simple clock is shown, displaying the current date and time.
- **User Not Identified.** It is shown when an unidentified user is standing in front of the mirror. In this page, the user can see their current avatar, the joybar, which displays their current emotional valence, and a simple message for the user to say if they want to initialize the registration process.
- **User Identified.** It is shown when the person in front of the mirror was identified. This page is the same as that of the unidentified user except the message displayed.
- **Registration.** It appears when the person in front of the mirror wants to register their identity in the system. This page guides the user through the registration phase, displaying what the user has to say to complete the process.
- **User’s personal page.** This page shows the weather for the user’s current location and other generic information. To access this page, the user is required not only to authenticate but also to show a positive emotional value in front of the mirror (for example, smiling). This is done not only as an initiative to ask the user to give a positive stimulus, but also as a therapeutic treatment for the user to be positive more often.

## 4. Experiments

In this section, we report the results of some experiments on the modules designed and built from scratch specifically for the system; these modules are speaker identification and speaker emotion recognition. For the other modules (i.e., those relating to visual interaction), we refer the reader to our previous papers [57,59].

The experiments were conducted on the Ryerson Audio-Visual Database of Emotional Speech and Song (RAVDESS) [72]. The database contains 1440 videos recorded from 24 professional actors (12 female, 12 male), vocalizing two lexically-matched statements in a neutral North American accent. There are eight different emotions including angry, calm, disgust, fearful, happy, neutral, sad, and surprise expressions. Each emotion apart from neutral is produced at two levels of emotional intensity (normal, strong). As a result, the neutral emotion is represented by 96 utterances, while the remaining emotions have the same number of utterances equal to 192. The metrics considered in our experiments are Precision, Recall, F1 score, and Accuracy.

### 4.1. Results for Speaker Identification

For validating the performance of the speaker identification model presented in Section 3.1.2 (Speaker Identification), we trained the *k*-NN classifier for categorizing the 24 actors of the RAVDESS dataset. We carried out experiments for speaker identification by performing 5-folds cross validation (cv). Table 5 reports the results in terms of Precision, Recall, F1-score, and Accuracy averaged over the 24 actors and the 5-cv iterations. We also compute the standard deviation on the 24 actors and 5-cv iterations. The achieved performance is very high (i.e., 99.97% of accuracy). This is motivated by the fact that the features extracted from the VoxCeleb1-trained network are highly discriminative, thus allowing the *k*-NN classifier to easily discriminate the different actors. Another aspect regards the content redundancy of the utterances, which permits the model to focus only on the speaker identity.

### 4.2. Results for Speaker Emotion Recognition

To evaluate the performance of the speaker emotion recognition model described in Section 3.1.2 (Speaker Emotion Recognition), we fine-tune the classification layer on the eight emotions of the RAVDESS dataset. For speaker emotion recognition, we investigated two scenarios: speaker-dependent and speaker-independent emotion recognition. For the speaker-dependent experiments, 5-fold cross-validation is performed using the same splits used for the assessment of speaker identification. In case of speaker independent experiments, Leave-One-Subject-Out (LOSO) test is performed. LOSO requires model to be trained with 1…(n−1) speakers and tested with *n*-th speaker. The process is repeated for each speaker.

#### Speaker-Dependent

**Figure 9:**
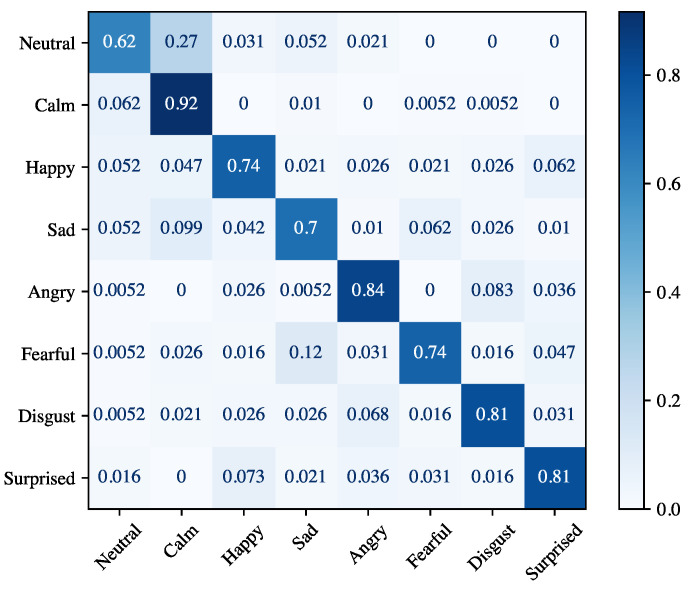
Confusion matrix for speaker-dependent emotion recognition.

Table 6 shows the results achieved for the speaker-dependent experiment. The performance for each emotion is measured separately, but we also report the average of the performance across all emotions. There are several considerations that can be made in the light of the results. Firstly, the average accuracy of 94.56% is 4% lower than that obtained for the speaker recognition task (99.97%), which indicates that discriminating emotions is a more complex task than recognizing the speaker. Secondly, among the different emotions, anger obtained the highest accuracy (95.47%), while the lowest accuracy was achieved by sad (92.91%). Thirdly, the relatively small standard deviation (1.69 on average) indicates that there are no substantial differences between the different cross-validation iterations. Figure 9 displays the confusion matrix for the speaker-dependent experiment. The worst accuracy was obtained for the class *neutral* (62%), which is mostly confused with the class *calm*. The latter is also the emotion achieving the best accuracy of 92%. Finally, the emotions that are often misclassified are *happy* vs. *surprised* and *angry* vs. *disgusted*, and vice versa.

#### Speaker-Independent

**Figure 10:**
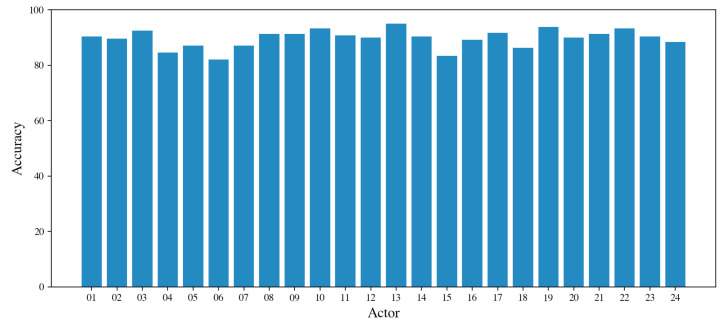
Speaker-independent emotion recognition. Accuracy achieved for each actor of RAVDESS dataset.

**Figure 11:**
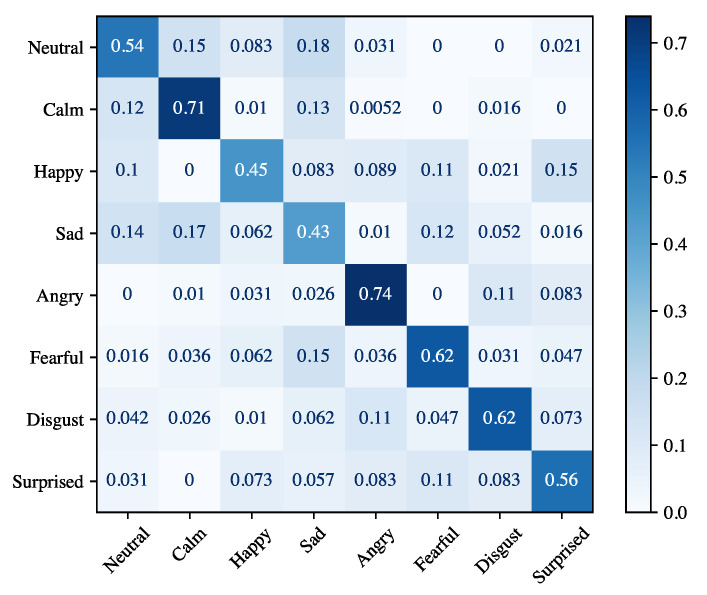
Confusion matrix for speaker-independent emotion recognition.

The results obtained for the speaker-independent emotion recognition are given in Table 7. As previously done for speaker-dependent experiments, we report both the performance for each emotion and the average across all emotions. We highlight that the achieved results are lower than those obtained for the speaker-dependent experiment. However, this was expected because in these experiments the classifier does not learn from the data of the test actor and is a more realistic scenario. The high variability of the performance for the different actors is also manifested in the very high value of the standard deviation (7.03% on average for the accuracy). An in-depth view of the accuracies obtained for each actor is given in Figure 10. Actors 13 and 19 achieve the highest accuracy (about 98%), while actors 04, 06, and 15 obtain the lowest, corresponding to about 82%. Figure 11 shows the confusion matrix for the speaker-independent experiment. We highlight that differently from the speaker-dependent experiment, the worst accuracy was achieved for the *sad* emotion (43%). It is mainly confused with *neutral* and *calm* emotions. On the other hand, *calm* and *angry* emotions obtain the best results with 71% and 74%, respectively.

### 4.3. System Usability Survey

We also performed a system usability study of the smart mirror. We conducted a survey with a panel of users to get feedback and opinions related the usability of the prototype. The survey was conducted within the University of Milano-Bicocca, and a total 20 people (10 males and 10 females) of ages between 22 and 33 participated in the survey. Each participant was asked to complete the registration phase, switch to the user’s personal page, use the daily reminder, and generally interact with the system. At the end of the test session, the user completed a survey based on the System Usability Scale (SUS) questionnaire [73]. The survey contains questions from the original SUS (questions 1–10), and questions tailored to investigate the smart mirror specific functionalities (questions 11–20). Specifically, the questions are:

1. I think I will often use this device.
2. I found this device too complex.
3. The device is easy to use.
4. I need the support from an expert to use the device.
5. I found each feature of the system well integrated.
6. There are too many inconsistency in this device.
7. I think the majority of the people will be able to quickly learn how to use this device.
8. I found this device really uncomfortable to use.
9. I felt at ease while using the device.
10. I had to learn many things prior starting to use the device.
11. I think the device is responsive.
12. I found the registration phase easy to complete.
13. I found redundant Alexa reading each written phrase on the mirror.
14. I think a touchscreen interaction would be useful for the mirror.
15. I found the conversation with the device natural and fluid.
16. The avatar represented on the mirror is what I look like.
17. I found easy to access the user’s personal page.
18. I found useful the reminder feature.
19. I think this device might violate my privacy.
20. I would use this device on my daily life.

**Figure 12:**
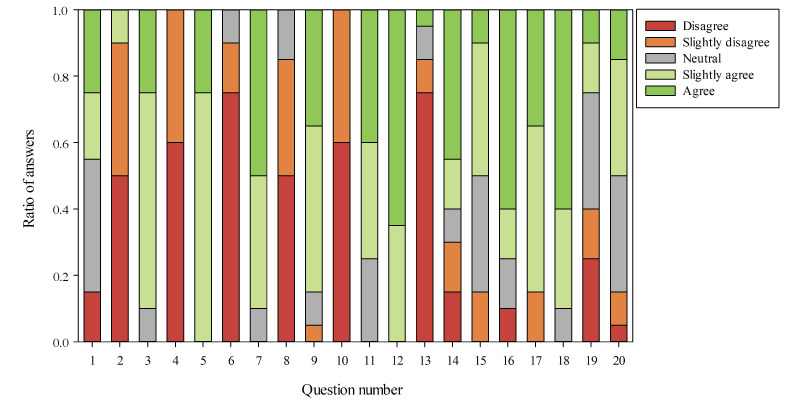
Results of survey indicating ratio of each answer for each question.

From the first 10 questions, a SUS score that represents the overall usability of the device, including how easy it is to use and learn, was calculated. The average SUS score, computed from the surveys of 500 products, is considered to be 68. Our smart mirror achieved 82.88, which is above average and in the range of good systems. In Figure 12, it is possible to view the users responses for each one of the 20 questions in the survey.

From the analysis of the responses, we can say that the smart mirror was well received overall. The users perceived the system as being very responsive and useful. Some users pointed out some problems with the Alexa service where the system could not fully understand the spoken name, which delayed the registration phase. This is probably due to the microphone used, which was not of sufficient quality for the environments in which the mirror was located. Another issue was found in the avatar creation. Although this feature was positively received, one female user complained that the system created a male avatar instead of a female one. A possible reason for this problem is that the data set used for the training of the facial attribute module contains mostly females with heavy makeup, thereby generating a bias during the training phase. A solution would be to train the module with a more diverse set of subjects. The users did not have any issues with the other functionalities.

### 4.4. Discussion

In this subsection, we summarize and comment on the experimental and technological results.

Experimental results for speaker-dependent and speaker-independent emotion recognition reported in Table 6 and Table 7 show that the proposed module can achieve high accuracy even if it did not learn emotional traits of the speaker (89.70% on average). The high standard deviation registered for precision, recall, and F1-score are not related to any bias in the dataset. In fact, the number of utterances for each emotion is practically identical. Regarding person-dependent experiments, the high standard deviation could be motivated by the fact that some emotions closely resemble each other. This is also evident in the confusion matrix in Figure 9. The *calm* emotion is often mistakenly classified with the *neutral* one. The *angry* emotion has many false positives (especially for the *disgust* emotion), and many false positives also occur for the *surprised* emotion. For the person-independent experiments, the lower performance of all the emotions is reasonable. In fact, each subject manifests their emotions in a different way, and therefore the emotion recognition model does not generalize well with new users.

Previous performance analysis shows that the developed recognition methods for audio emotion recognition are effective. In general, the modules developed for video and audio interaction represented the state-of-the-art at the time of the development of the mirror. More robust methods for both video and audio identification were recently proposed [74,75]. At the same time, more effective face attribute estimation [76] and speaker emotion recognition [77,78] methods were presented. Therefore, to meet the growing security needs and improve the user experience, we plan to further improve our methods in the future.

From a technical point of view, since the mirror is used in domestic environments, we developed it to have low power consumption. As previously described, heavy computing is dedicated to an external server, devoting the smart mirror almost exclusively to I/O operations. Thus, the resulting power consumption of the mirror relies only on the Raspberry and the display monitor. When the mirror is in the idle state, the power consumption is approximately 23 W. This consumption is split between 15–20 W for the display monitor (that is always on), and 2.6–2.8 W for the Raspberry (including connected devices). In the working state, the power consumption increases to about 25 W. The consumption of the Raspberry rises to 4.7–4.8 W, while that of the display monitor is unchanged.

## 5. Conclusions

In this paper, we described the design of a prototype smart mirror that is capable of interacting with the current user in different modalities and is able to recognize their emotions from visual and audio data. The mirror exploits deep learning techniques to implement the relevant tasks associated with user identification, facial attribute detection, and emotion recognition.

Our smart mirror prototype incorporates many functionalities that other smart mirrors found in the literature, both academic and commercial, do not possess. Most of state-of-art smart mirrors are simple, with only few functionalities, or are limited by the computational capability of the on-board processing devices. We designed our smart mirror by including an external server and separating the workload between the on-board device and the server. This led to the possibility of including many features that normally require a lot of computational power, while decreasing the computation time at the same time. Also, the interaction with Amazon Alexa is another strength of this prototype. Firstly, having a virtual assistant that allows a dialogue close to the natural language improves the interaction with the mirror, especially for those users that are not used to technology, such as elders. Secondly, by exploiting this service, we further reduce the computational workload since every skill resides on the Amazon Web Service.

Following the users’ feedback and interactions with the mirror during usability tests, hardware improvement is under investigation. For example, to increase the image quality even with poor lightning, a night vision camera could be added to the mirror. We are currently devising how to incorporate this within the processing workflow. Also the microphone will be replaced with a better performing one. The data provided from the emotion recognition features are important for monitoring and understanding the user’s long-term emotional progress. At the moment, this feature is under development, and it will be integrated via an ad-hoc data management module and visual application to browse and analyze the recorded data.

One of the limitations of the proposed prototype is that it is a proof of concept experimentally validated in a laboratory environment. A future development could be the deployment of the proposed prototype in a real-world environment to increase the technology-readiness of the system. To this end, we are considering the use of a development board, such as the NVIDIA Jetson Family Boards (https://www.nvidia.com/en-gb/autonomous-machines/embedded-systems/ (accessed on 15 October 2021)), with the aim of reducing as much as possible the use of costly server-side computational power and data exchange. This will require a suitable re-engineering of the recognition modules since development boards have limited computational capacity with respect to traditional servers [79]. 

## Figures and Tables

**Table 3:** List of 40 face attributes provided with CelebA database [60].

Index	Attribute	Index	Attribute	Index	Attribute	Index	Attribute
1	5o’Clock Shadow	11	Blurry	21	Male	31	Sideburns
2	Arched Eyebrows	12	Brown Hair	22	Mouth Slightly Open	32	Smiling
3	Attractive	13	Bushy Eyebrows	23	Mustache	33	Straight Hair
4	Bags Under Eyes	14	Chubby	24	Narrow Eyes	34	Wavy Hair
5	Bald	15	Double Chin	25	No Beard	35	Wearing Earrings
6	Bangs	16	Eyeglasses	26	Oval Face	36	Wearing Hat
7	Big Lips	17	Goatee	27	Pale Skin	37	Wearing Lipstick
8	Big Nose	18	Gray Hair	28	Pointy Nose	38	Wearing Necklace
9	Black Hair	19	Heavy Makeup	29	Receding Hairline	39	Wearing Necktie
10	Blond Hair	20	High Cheekbones	30	Rosy Cheeks	40	Young

**Table 4:** Table containing basic information regarding datasets considered for training of the speaker emotion recognition method.

	CREMA-D	EMO-DB	SAVEE	TESS
# samples	7442	535	480	2800
# actors	91	10	4	2
# male	48	5	4	0
# female	43	5	0	2
# of Emotions	6	7	7	7
Age Range	20–74	21–35	27–31	26–64
Actors first Language	English	German	English	English
Actors Origin Accent	Multiethnic	Germany	South-East England	North American
Sample rate	16,000	16,000	44,100	24,414

**Table 5:** Speaker identification performance. Average (±standard deviation) precision, recall, F1-score, and accuracy on the 24 actors of RAVDESS dataset.

	Precision	Recall	F1-Score	Accuracy
Average	99.70 ± 1.70	99.65 ± 2.06	99.66 ± 1.48	99.97 ± 0.12

**Table 6:** Speaker-dependent emotion recognition. Average (±standard deviation) precision, recall, F1-score, and accuracy across 5-cv iterations. In each column, the best results are marked in **boldface**.

	Precision	Recall	F1-Score	Accuracy
Angry	85.63 ± 13.02	84.24 ± 5.84	**84.09** ± 5.78	**95.47** ± 2.32
Calm	74.23 ± 6.06	**91.78** ± 5.00	81.74 ± 2.62	94.51 ± 0.99
Disgust	83.68 ± 7.52	80.26 ± 7.27	81.42 ± 3.50	95.14 ± 0.97
Fearful	**86.14** ± 9.13	73.55 ± 15.43	77.70 ± 9.45	94.72 ± 1.53
Happy	80.47 ± 9.72	74.63 ± 7.94	76.84 ± 6.02	93.97 ± 1.65
Neutral	62.29 ± 2.77	61.97 ± 13.93	61.11 ± 6.92	94.88 ± 1.50
Sad	77.02 ± 9.51	69.70 ± 14.71	71.44 ± 7.28	92.91 ± 0.97
Surprised	83.49 ± 12.96	79.22 ± 17.13	78.89 ± 10.65	94.91 ± 1.71
Average	79.12 ± 12.00	76.92 ± 14.58	76.65 ± 9.82	94.56 ± 1.69

**Table 7:** Speaker-independent emotion recognition. Average (±standard deviation) precision, recall, F1-score, and accuracy across 24 actors of RAVDESS dataset. In each column, the best results are marked in **boldface**.

	Precision	Recall	F1-Score	Accuracy
Angry	78.64 ± 20.78	**73.96** ± 24.45	**70.29** ± 19.91	91.81 ± 5.83
Calm	**79.18** ± 19.48	71.35 ± 30.93	66.38 ± 24.95	**91.94** ± 4.21
Disgust	69.70 ± 23.63	62.50 ± 28.64	61.28 ± 23.24	90.83 ± 3.97
Fearful	74.10 ± 22.93	62.50 ± 27.24	60.86 ± 22.53	89.86 ± 7.13
Happy	77.35 ± 26.44	44.79 ± 29.52	47.30 ± 24.14	88.75 ± 4.60
Neutral	50.44 ± 35.06	54.17 ± 41.25	40.22 ± 30.81	90.83 ± 6.70
Sad	61.74 ± 33.27	42.71 ± 30.39	39.58 ± 23.84	84.44 ± 11.35
Surprised	73.74 ± 23.39	56.25 ± 30.41	55.64 ± 20.91	89.17 ± 6.38
Average	70.61 ± 27.77	58.53 ± 32.47	55.19 ± 26.35	89.70 ± 7.03

## Data Availability

The RAVDESS dataset can be found at https://zenodo.org/record/1188976#.YYoz6C9aZAY (accessed on 15 October 2021).
